# Social and cognitive factors influencing commercial chicken farmers’ antimicrobial usage in Bangladesh

**DOI:** 10.1038/s41598-022-26859-8

**Published:** 2023-01-11

**Authors:** Tasneem Imam, Justine S. Gibson, Suman Das Gupta, Mohammad Foysal, Shetu B. Das, Md Ahasanul Hoque, Guillaume Fournié, Joerg Henning

**Affiliations:** 1 The University of Queensland, Gatton, QLD Australia; 2 Chattogram Veterinary and Animal Sciences University, Chattogram, Bangladesh; 3 Royal Veterinary College, London, UK

**Keywords:** Risk factors, Environmental social sciences, Sustainability

## Abstract

Adapting the Social Cognitive Theory framework, we conducted a cross-sectional study on 137 commercial chicken farms in Bangladesh to investigate factors influencing the *behaviour* of farmers towards the application of antimicrobials to their birds. Almost all farmers used antimicrobials to treat poultry diseases, while 38.6% also were using them to promote healthy growth of chickens and 10.2% to increase egg production or improve meat quality. Using Structural Equation Modeling (SEM), we identified that inappropriate usage of antimicrobials (*behaviour*) was strongly driven by farmers’ short-term *goals* to maintain the health of their chickens in a production cycle (β = 0.813, p = 0.029), rather than long-term concerns. Farmers’ perception about their ability to control antimicrobial administration based on their skills and opportunities (*self-efficacy*) marginally influenced the short-term *goals* of antimicrobial usage (β = 0.301, p = 0.073). The results of this study can be used to develop targeted education programs for farmers, to reduce the application of antimicrobials in their poultry flocks.

## Introduction

Antimicrobial resistance is considered as a global threat to human health^1^ and action plans to tackle this problem have been developed by the World Health Organisation (WHO)^2^. To investigate the awareness towards antimicrobial resistance among public health and agriculture experts as well as policymakers, WHO conducted a survey of 9772 participants from 12 countries (China, Vietnam, India, Indonesia, Egypt, Sudan, Russian Federation, Siberia, Barbados, Mexico, Nigeria and South Africa) between September and October 2015^3^. About 57% of the respondents indicated that ‘there is not much people like them can do to stop resistance development’ and 44% believed that ‘resistance is only a problem for those who take antimicrobials regularly’. The report also highlighted that people of lower income countries are less aware of antimicrobial resistance compared to people of higher income countries^3^.

Inappropriate use of antimicrobials in food animals has contributed to the emergence of antimicrobial resistance^4^. Misconceptions about antimicrobial usage are common among farmers, and disease occurrence due to poor biosecurity^5^ and a lack of strategic vaccinations^6^ might influence farmers’ behaviour towards antimicrobial applications. For example, some farmers believe that antimicrobials could improve the immunity of chickens and, that antimicrobial usage for disease prevention or growth promotion may not result in antimicrobial resistance^7^. Furthermore, some farmers believe that antimicrobials should be administered without veterinarians’ advice^7^ and that preventive usage of antimicrobial is more important than improving biosecurity^8^. Other perceptions of farmers are that antimicrobials can be prescribed by traders^9^, that the use of multiple antimicrobials is important to control diseases on farms^7^, that antimicrobial usage on food animals does not have any impact on human health^10^ and that antimicrobials can be used without adhering to withdrawal periods^11^.

The decision-making process of farmers to implement or to not implement appropriate management practices is complex and different approaches have been used to analyse farmer’s behaviours and the factors associated with these behaviours. The knowledge, attitude, and practice (KAP) approach has been applied in the context of antimicrobial usage^7,12,13^. KAP studies are popular as they are easy to design, less time consuming and less costly than in-depth qualitative studies^14,15^. However, KAP approaches has been criticized by social scientists as the behaviour of a person represents interlinked characteristics of this person’s knowledge, beliefs, emotions and values, which are not as easily captured in responses to separate individual questions in a KAP questionnaire^15^. Furthermore, knowledge (which is a key component evaluated in KAP studies) is only one of many factors that influence how people seek to address a problem; thus, a direct relationship between knowledge and behaviour cannot be assumed. To change behaviour, extension and intervention programmes need to address additional factors ranging from sociocultural to environmental and economic components, which are usually not captured in KAP studies^16–20^.

On the other hand, theoretical concepts such as the Health Belief Model^21^, Theory of Reasoned Action^22^, Theory of Planned Behaviour^23^ and Protection Motivation Theory^24^ and Social Cognitive Theory^25,26^ represent applied psychological frameworks that do allow to comprehensively analyse behaviours and factors influencing them^25^. The ‘Social Cognitive Theory’ in particular has been used to describe social and cognitive factors that impact human behaviour^25,26^. This framework has also been used to study populations in which interventions of ‘healthier habits’ were introduced. For example, it has been applied to describe how technological innovations can change the behaviour of diabetic people^25^, how vocational services for people with psychiatric disorders can be improved^27^, how web-based learning systems for students can be enhanced^28^, and how behaviour relating to physical activities can be improved^29^. This framework has also been applied to investigate farmers’ behaviour towards water conservation^30^ and to explore the usage of climate forecasts to make decisions about crop management^31^.

Therefore, we considered the ‘Social Cognitive Theory’ as a flexible and applied psychological framework to evaluate the behaviour of commercial farmers towards administration of antimicrobials in their chicken flocks.

## Results

The study population included 137 commercial layer and broiler chicken farmers operating in Chattogram, Bangladesh, with 83 farmers raising broiler chickens and 54 laying hens. Most broiler (98.8%, 82/83) and all layer farmers (100.0%, 54/54) were male. Most layer farmers (61.1%, 33/54) and about half of the broiler farmers (49.4%, 41/83) had ≥ 10 years of farming experience. More layer famers (92.6%, 50/54) had a secondary level of education compared to broiler farmers (78.3%, 65/83)^32^.

Frequency statistics of all the responses collected on a five-point Likert scale for each ‘observed variable’ under the ‘latent constructs’ are presented in Table S1, while the frequency statistics of the responses for each ‘observed variable’ maintained in the final Structural Equation Model (SEM) under the ‘latent constructs’ (*behaviour*, *self*-*efficacy* and *goals*) are shown in Table 1. The *behaviour* of farmers to use antimicrobials in their chicken flocks was the outcome ‘latent construct'.

**Table 1:** Percentage (N) of responses to statements (‘observed variables’) provided by commercial layer and broiler chicken farmers in Chattogram, Bangladesh.

Statement	Strongly disagree	Disagree	Do not know	Agree	Strongly agree
	% (N)	% (N)	% (N)	% (N)	% (N)
**Behaviour**					
I am increasing the dosage of antimicrobials when I am experiencing more chicken getting sick or dying (Beh1)*	5.1 (7)	43.8 (60)	0.0 (0)	48.9 (67)	2.2 (3)
I always have a range of antimicrobials available on my farm, even if I do not used them all (Beh3)*	5.8 (8)	61.3 (84)	0.0 (0)	32.8 (45)	0.0 (0)
**Self-efficacy**					
I believe that stronger laws and enforcement of the law are needed to reduce antimicrobial usage (SEff3)	0.0 (0)	0.0 (0)	5.1 (7)	47.4 (65)	47.4 (65)
I would invest time and money to further improve farm hygiene and biosecurity to reduce the usage of antimicrobial on my farm (SEff4)	0.0 (0)	10.9 (15)	4.4 (6)	64.2 (88)	20.4 (28)
**Goals**					
Antimicrobials lead to a healthy growth of chickens (Goal1)*	3.6 (5)	55.5 (76)	2.2 (3)	32.8 (45)	5.8 (8)
Antimicrobials help chickens to recover from disease (Goal2)*	0.0 (0)	0.0 (0)	0.7 (1)	87.6 (120)	11.7 (16)
Antimicrobials help increasing egg production or improving the quality of the chicken meat (Goal3)*	5.1 (7)	73.0 (100)	11.7 (16)	8.0 (11)	2.2 (3)

Abbreviations in brackets (Beh, SEff and Goal) represent individual statements in the questionnaire for which responses were captured in the interview (see Table S1 for more details).

*Recoded for analysis.

### Behaviour

About half (51.1%, 70/137) of farmers either agreed or strongly agreed that they used an increased dose of antimicrobials when they observed more chicken getting sick or dying (Table 1). About a third of farmers (32.8%, 45/137) acknowledged to stock a range of antimicrobials on their farms even if there was no need to use them.

### Self-efficacy

The majority of farmers (94.8%, 130/137) indicated that an enforcement of stronger laws is needed to reduce antimicrobial usage. Most farmers (84.6%, 116/137) also indicated that they would invest time and money to further improve farm hygiene and biosecurity to reduce the usage of antimicrobials on their farms.

### Goals

Almost all farmers (99.3%, 136/137) highlighted that antimicrobials help chickens to recover from disease. About a third (38.6%, 53/137) of farmers mentioned that antimicrobials promote a healthy growth of chickens, while a small proportion of them (10.2%, 14/137) indicated that antimicrobials help to increase the egg production or improve the quality of the chicken meat.

## Structural Equation Modeling (SEM)

Using confirmatory factor analysis (CFA) in the measurement part of the SEM, no significant association was identified for any of the ‘observed variables’ with the latent construct *socio-structural factors* (p > 0.05). Therefore, the latent construct *socio-structural factors* was not included in the structural part of the SEM.

Using path analysis in the structural part of the SEM, the latent constructs *outcome expectations*, *goals*, *self-efficacy*, and *behaviour* (as the outcome variable) were considered. The latent construct *outcome expectations* did not significantly (p = 0.501) influence farmers’ *behaviour* to use antimicrobials on their farms and was therefore excluded.

**Figure 1:**
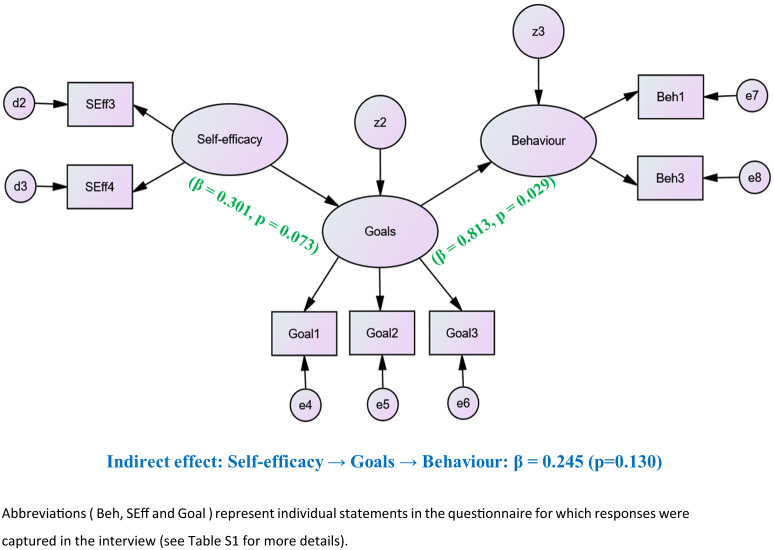
Final Structural Equation Model describing farmers’ *behaviour* towards antimicrobial administration on commercial chicken farms in Chattogram, Bangladesh that is based on the ‘Social Cognitive Theory’ framework^25^. ‘Observed variables’ are presented in rectangles and the ‘latent constructs’ are presented in ovals. Both ‘d’ and ‘e’ represent errors of measurements, while ‘z’ represents the residuals of the latent constructs.

The final SEM path is shown in Fig. 1. The results indicate that *behaviour* of commercial chicken farmers to increase the usage of antimicrobials or to have a range of antimicrobials available for usage on farms, increased with their (short-term) *goals* to improve the health of their chickens (β = 0.813, p = 0.029). *Self-efficacy* had a marginal impact on the goals of farmers to improve the health of their chickens (β = 0.301, p = 0.073), and had no significant direct impact on *behaviour* of farmers related to antimicrobial usage.

Overall, the data fit the model well (χ^2^ = 8.724, p = 0.647; RMSEA < 0.01, CFI = 1.000, SRMR = 0.045).

## Discussion

To the best of our knowledge, this is the first published research study that used the ‘Social Cognitive Theory’ framework to explore social and cognitive factors influencing farmers’ *behaviour* towards antimicrobial administration on commercial chicken farms.

Overall, this study found that farmers’ *behaviour* is primarily directed by their behavioural *goals*. Commercial chicken farmers were concerned about disease occurrence in their chicken flocks, and farmers’ *goals* to maintain the health of their chickens was driving their use of antimicrobials. These results may explain the large number of antimicrobials applied on these farms^32^ and might help to eludicate similar high usages reported from other Asian countries such as India, Nepal, Thailand, China and Sri Lanka^33^.

Bandura highlighted that individual *goals* are considered ‘effective’ in adapting habits. For example, *goals* were the most important determinant in developing healthy *behaviours* such as stopping smoking, reducing weight and performing exercise^34^. Our study highlighted that farmers created ‘short-term’ attainable *goals*^25^, by focussing mainly on poultry health outcomes and thereby immediate benefits in the current production cycle. Indeed, recurrent beneficial feedback^26^ from antimicrobial administration over multiple production cycles might have ‘psychologically’ shaped farmers’ *behaviour* towards inappropriate antimicrobial application. On the contrary, people only tend to change their behaviour when the outcome of their behavioural set *goals* is dissatisfactory^35^. Thus, the administration of antimicrobials could be considered as a ‘comfortable and convenient' solution for farmers (which might therefore be a *behaviour* that farmers are unwilling to change) as antimicrobials are readily available over-the-counter without a prescription^33^ or directly through feed and chick traders^9^ while being easily applied to chickens through feed or drinking water^36^. Farmers’ intentions of executing rather ‘short-term’ *goals* have been also illustrated by the fact, that farmers’ responses under *outcome expectations*, which represent more ‘long-term’ goals (and concerns), did not influence their behavioural pattern towards antimicrobial usage.

According to the ‘Social Cognitive Theory’, *goals* are determined by *self-efficacy*^25^. In consistency with this theory, we found that *self-efficacy* marginally impacted farmers’ *goals* regarding antimicrobial application. It has been described previously that *self-efficacy* influences peoples’ thinking^37^ and thereby their *goal* setting to demonstrate an actual behaviour^38,39^. We found that most farmers were willing to invest time and money to improve farm biosecurity and they were also in support of strict laws to limit antimicrobial usage.

Previous research has highlighted that poultry farmers in Bangladesh are unable to control disease occurrences themselves through the administration of antimicrobials^40^, but also that antimicrobials are frequently administered to chickens in the absence of clinical signs (24.8% of farms) and without adhering to withholding periods (83.3% of layer and 36.1% of broiler farms)^32^. Therefore, farmers ability to perform a desired *behaviour*, of reduced antimicrobial usage, requires adequate training, demonstration, and reinforcement^25^.

We did not identify ‘observed variables’ that significantly influenced the latent construct *‘socio-structural factors'*, which was therefore not included in the final model. It could have been the case that *‘socio-structural factors'* were not sufficiently described by the recorded ‘observed variables and other variables might need to be considered in future research. For example, *‘socio-structural factors'* might be related to the availability of vaccinations for chickens^41^, the influence of representatives from pharmaceutical companies on farmers^9^, lack of financial capital^42^ or the opinions of neighbouring farmers^43^. Also, due to the cross-sectional nature of this research, we could not confirm (for example through observations) the reported *behaviour* of farmers, so a validation of the hypothesized causal relationships between ‘latent constructs' could not be performed. A qualitative data collection approach with in-depth interviews would be helpful to explore the identified behaviour of farmers in more detail.

Overall, the research presented here highlighted the short-term *goal* oriented *behaviour* of commercial poultry farmers in Bangladesh. These observations are valuable for policy makers for designing extension programs aiming to implement *behaviour* changes in regard to antimicrobial administration. However, *behaviours* of individuals are generally difficult to modify^44^ and innovative strategies are required. WHO has developed a guide for Tailoring Antimicrobial Resistance Programmes (TAP) in order to determine perceived barriers and drivers of behaviour change^45,46^. Behavioural insights specialists working within the TAP highlighted the importance of cultural and social contexts for changing the behaviour of target populations^45^. Lessons from the TAP are useful for designing programs to change the behaviour of poultry farmers in Bangladesh. For example, farmers are less likely to know the generic names of antimicrobials and they are more familiar with the trade names^7^. Therefore, it is important that extension programs consider the knowledge and social background of Bangladeshi poultry farmers, and that effective and cultural-sensitive communication approaches are developed and applied. There are some existing initiatives in Bangladesh under which training of poultry farmers could be delivered. For example, the Department of Livestock Services (DLS) has set-up the Upazila to Community (U2C) initiative, which aims to empower women in rural communities to improve livestock production and disease control ^47^. Furthermore, the Bangladesh AMR Response Alliance (BARA) was created to involve both government agencies and private health professionals to ensure responsible use of antimicrobials at the community level^47^. Tapping into these existing community networks would provide opportunities to deliver training on poultry diseases, biosecurity practices and antimicrobial usage, and overall improve poultry production and might be helpful to change the short-term *goal* oriented *behaviour* of commercial chicken farmers.

## Materials and methods

### The ‘Social Cognitive Theory’ framework

**Figure 2:**
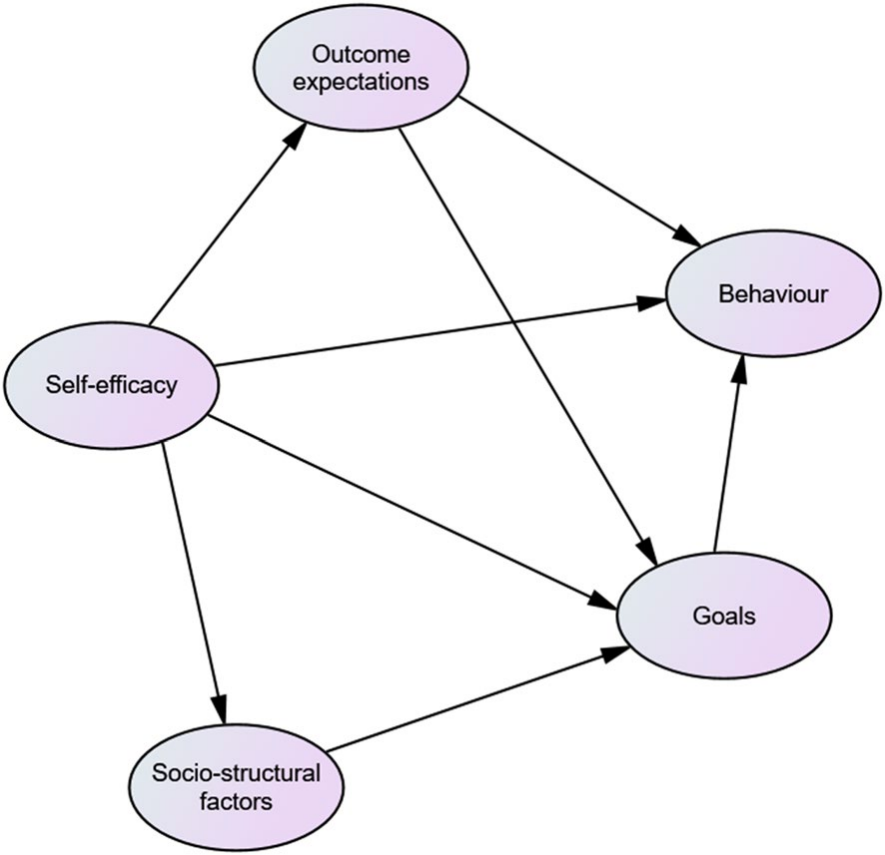
Hypothesized Structural Equation Modeling paths describing farmers’ *behaviour* towards the administration of antimicrobials in commercial chicken flocks in Chattogram, Bangladesh.

The ‘factors’ or components of the ‘Social Cognitive Theory’ framework are *self-efficacy*, *goals*, *outcome expectations* and *socio-structural factors,* which directly or indirectly influence *behaviour*^25^. Figure 2 depicts the hypothesized paths or relationships between individual factors and how they regulate, or impact *behaviour* as described by Bandura in 2004^25^.

While *self-efficacy* measures the ability of people to successfully overcome challenges to perform a *behaviour*, *outcome expectations* measure the expected favourable and unfavourable effects of the *behaviour* including positive and negative self-evaluative reactions^25^. *Goals*, which include short-term attainable objectives guide people’s actions^25^, while *socio-structural factors* represent the perceived facilitators and obstacles that influence a *behaviour*^25^. Bandura emphasized the importance of *self-efficacy* to directly influence *behaviour* of humans, but also to influence the other factors^25^.

We have conceptualized these ‘factors’ of the ‘Social Cognitive Theory’ in relation to farmer’s *behaviour* in administering antimicrobials to their chicken flocks and defined these ‘factors’ as follows:

1. Perceived *self-efficacy* relates to the belief of farmers that they could control the usage of antimicrobials based on their own assessment of their skills and opportunities. For example, the statement ‘I believe that stronger laws and enforcement of the law are needed to reduce antimicrobial usage’ belongs to *self-efficacy*.
2. *Outcome expectations* relates to farmers’ perceived benefits from using antimicrobials and the effect that antimicrobial administration will have on poultry health, production and human health. An example for an *outcome expectation* would be the statement ‘Antimicrobial residues in chicken meat will not harm humans’.
3. *Goals* represent achievable short-term objectives that encourage farmers to administer antimicrobials. ‘Antimicrobials help to increase egg production or improve the quality of the chicken meat’ is an example statement for *Goals*.
4. *Socio-structural factors* are perceived external facilitators and impediments that encourage or deter farmers to use antimicrobials. ‘I am bound to take advice from feed traders because I owe them money (they provide day old chicks, antimicrobials, and feed)’ illustrates a statement for *socio-structural factors*.

### Study design

A cross-sectional study was used to collect data on farmers’ usage and perception of administering antimicrobials to their layer and broiler chicken flocks in the Chattogram district of Bangladesh. The Chattogram district was selected because it is a centre for commercial chicken production in Bangladesh^48^.

First, a sampling frame of 1,748 commercial chicken farms in this district was developed with the help of the Bangladesh District Livestock Services (DLS), feed and chick traders, pharmaceutical representatives, and government and private practitioners^49^. From this sampling frame, 140 commercial chicken farmers from 8 *upazilas* (sub-districts) were selected using simple random sampling (using syntax RANDBETWEEN in Microsoft Excel). Farmers were interviewed between February and May 2019 and 137 of these 140 farmers reported of using antimicrobials and these 137 farmers were in the further analysis. Further details about the sampling approach are provided in^32^.

### Questionnaire

A structured questionnaire was developed to collect data on ‘factors’ of the ‘Social Cognitive Theory’ framework. Each ‘factor’ was evaluated by a series of statements (’observed variables’) for which farmers provided responses on a 5-point Likert scale: ‘Strongly disagree’, ‘Disagree’, ‘Do not know’, ‘Agree’, and ‘Strongly agree’.

### Data analysis

Structural Equation Modeling (SEM) was used to analyse the dataset. The SEM is comprised of two parts, a measurement and a structural part^50^. In the measurement part of the SEM, statements (or ‘observed variables’) are used to build each of the separate ‘factors’ according to ‘Social Cognitive Theory’. These ‘factors’ are termed ‘latent constructs’ in SEMs. Confirmatory Factor Analysis (CFA) was then applied to identify which of the ‘observed variables’ would be included in each ‘latent construct’. In the structural part of the SEM, path analysis was used to describe the relationship between the causal ‘latent constructs’ (i.e. *self-efficacy*, *outcome expectations, goals,* and *socio-structural factors*) and how they impacted the outcome ‘latent construct’ *behaviour* (which represented the behaviour of farmers towards antimicrobial usage on their farms). To ensure all ‘observed variables’ are scaled in the same direction^51^, some of the original responses were recoded. The conceptual framework with all collected ‘observed variables’ informing each ‘latent construct’ and the relationships between ‘latent constructs’ is displayed in Fig. S1.

A p-value ≤ 0.05 was selected as cut-off to include ‘observed variables’ under each of the ‘latent constructs’ in the CFA and a p-value ≤ 0.1 was selected as cut-off to maintain ‘latent constructs’ in the path analysis.

The overall model fit was assessed by the chi-square (χ)^2^ statistic with a p-value < 0.05 as an indicator of good fit^52^. The root mean square error of approximations (RMSEA) was also used, with values < 0.05 indicating a good fit and values up to 0.08 indicating an acceptable fit^53^. Furthermore, the comparative fit index (CFI) with values > 0.95 indicating very good fit and ≥ 0.90 an acceptable fit^52^ was also applied. In addition, standard root mean square residuals (SRMR) values ≤ 0.05 were considered indicative of a close-fitting model while values between 0.05 up to 0.10 were suggesting acceptable fit^54^.

Descriptive data analysis was conducted in STATA 16 (StataCorp®, 2019) while the SEM was developed using AMOS 27 (IBM® SPSS® Amos™ 27, 2020).

### Ethics approval

Human Ethics Approval for the interviews was obtained from the University of Queensland Institutional Human Ethics Committee on the 7 December 2018 (Approval number: 2018002266). The outlined research with farmers was carried out in accordance with relevant guidelines and regulations (Declaration of Helsinki) and informed consent was obtained from all participants (none of the participants was under 18 years of age).

## Supplementary Information

Supplementary Information.

## Data Availability

The raw data supporting the conclusions of this research will be made available upon request by the first author of this publication, Tasneem Imam (t.imam@uq.edu.au).

## References

[1] Balsalobre LC, Dropa M, Matté MH (2014). An overview of antimicrobial resistance and its public health significance. Braz. J. Microbiol..

[2] WHO (2015). Global Action Plan on Antimicrobial Resistance.

[3] WHO (2015). Antibiotic Resistance: Multi-country Public Awareness Survey. Report No. 9241509813.

[4] Löhren U, Ricci A, Cummings TS, Guardabassi L, Jensen LB, Kruse H (2008). Guide to Antimicrobial Use in Animals.

[5] Raasch S, Postma M, Dewulf J, Stärk KDC, Grosse Beilage E (2018). Association between antimicrobial usage, biosecurity measures as well as farm performance in German farrow-to-finish farms. Porc. Health Manag..

[6] Buchy P (2020). Impact of vaccines on antimicrobial resistance. Int. J. Infect. Dis..

[7] Nuangmek A (2018). Knowledge, attitudes and practices toward antimicrobial usage: A cross-sectional study of layer and pig farm owners/managers in Chiang Mai, Lamphun, and Chonburi provinces, Thailand. Korean J. Vet. Res..

[8] Rimi NA (2017). Biosecurity conditions in small commercial chicken farms, Bangladesh 2011–2012. EcoHealth.

[9] Masud AA (2020). Drivers of antibiotic use in poultry production in Bangladesh: Dependencies and dynamics of a patron-client relationship. Front. Vet. Sci..

[10] Di Martino G (2019). Farmers' attitudes towards antimicrobial use and awareness of antimicrobial resistance: A comparative study among turkey and rabbit farmers. Ital. J. Anim. Sci..

[11] Xu J, Sangthong R, McNeil E, Tang R, Chongsuvivatwong V (2020). Antibiotic use in chicken farms in northwestern China. Antimicrob. Resis. Infect. Control..

[12] Hu Y (2018). Knowledge, attitude, and practice with respect to antibiotic use among Chinese medical students: A multicentre cross-sectional study. Int. J. Environ. Res. Public Health..

[13] Kalam MA (2022). Knowledge, attitudes, and common practices of livestock and poultry veterinary practitioners regarding the AMU and AMR in Bangladesh. Antibiotics.

[14] Stone L, Campbell J (1984). The use and misuse of surveys in international development: An experiment from Nepal. Hum. Organ..

[15] Launiala A (2009). How much can a KAP survey tell us about people's knowledge, attitudes and practices? Some observations from medical anthropology research on malaria in pregnancy in Malawi. Anthrop. Matters..

[16] Smith H (1993). On the limited utility of KAP-style survey data in the practical epidemiology of AIDS, with reference to the AIDS epidemic in Chile. Health Transit. Rev..

[17] Cleland J (1973). A critique of KAP studies and some suggestions for their improvement. Stud. Fam. Plann..

[18] Future Learn. *The Shortcoming of KAP Studies*. https://www.futurelearn.com/info/courses/one-health/0/steps/25495 (accessed on 30 June 2021).

[19] University of Bristol. *The Shortcoming of KAP Studies*. https://tales.nmc.unibas.ch/en/one-health-connecting-humans-animals-and-the-environment-13/one-health-qualitative-and-mixed-methods-61/the-shortcoming-of-kap-studies-437 (accessed on 30 June 2021).

[20] Andrade C, Menon V, Ameen S, Kumar Praharaj S (2020). Designing and conducting knowledge, attitude, and practice surveys in psychiatry: Practical guidance. Indian J. Psychol. Med..

[21] Champion VL, Skinner CS (2008). Health Behavior and Health Education: Theory, Research, and Practice.

[22] Montaño DE, Kasprzyk D (2015). Health Behavior and Health Education: Theory, Research, and Practice.

[23] Ajzen I (2011). The theory of planned behaviour: Reactions and reflections. Psychol. Health..

[24] Norman P, Boer H, Seydel ER (2005). Predicting Health Behaviour.

[25] Bandura A (2004). Health promotion by social cognitive means. Health Educ. Behav..

[26] Bandura A (1986). Social Foundations of Thought and Action: A Social Cognitive Theory.

[27] Fabian ES (2000). Social cognitive theory of careers and individuals with serious mental health disorders: Implications for psychiatric rehabilitation programs. Psychiatr. Rehabil. J..

[28] Wang SL, Lin SS (2007). The application of social cognitive theory to web-based learning through *NetPorts*. Br. J. Educ. Technol..

[29] Oyibo K, Adaji I, Vassileva J (2018). Social cognitive determinants of exercise behavior in the context of behavior modeling: A mixed method approach. Digit. Health.

[30] Yazdanpanah M, Feyzabad FR, Forouzani M, Mohammadzadeh S, Burton RJ (2015). Predicting farmers’ water conservation goals and behavior in Iran: A test of social cognitive theory. Land Use Policy.

[31] Jaberi Z, Baradaran M, Yazdanpanah M (2019). Analysis the role of Psychological factors on intention to apply environmental and meteorological information by farmers in Dehloran Town (The combined application of social cognition theory and technology acceptance Model). J. Environ Stud..

[32] Imam T (2020). A cross-sectional study of antimicrobial usage on commercial broiler and layer chicken farms in Bangladesh. Front. Vet. Sci..

[33] Goutard FL (2017). Antimicrobial policy interventions in food animal production in South East Asia. BMJ.

[34] Alexy B (1985). Goal setting and health risk reduction. Nurs. Res..

[35] Wood RE, Mento AJ, Locke EA (1987). Task Complexity as a moderator of goal effects: A meta-analysis. J. Appl. Psychol..

[36] Diarra MS, Malouin F (2014). Antibiotics in Canadian poultry productions and anticipated alternatives. Front. Microbiol..

[37] Bandura A, Wood R (1989). Effect of perceived controllability and performance standards on self-regulation of complex decision making. J. Pers. Soc. Psychol..

[38] Holman H, Lorig K (1992). Perceived self-efficacy in self-management of chronic disease. Self-efficacy.

[39] Plotnikoff RC, Lippke S, Courneya KS, Birkett N, Sigal RJ (2008). Physical activity and Social Cognitive Theory: A test in a population sample of adults with type 1 or type 2 diabetes. Appl. Psychol..

[40] Kabir SML (2016). Prevalence of poultry diseases in Gazipur district of Bangladesh. Asian J. Med. Biol. Res..

[41] Alders R (2007). Challenges and constraints to vaccination in developing countries. Dev. Biol. (Basel).

[42] Mengesha M (2013). Biophysical and the socio-economics of chicken production. Afr. J. Agric. Res..

[43] Manyi-Loh C, Mamphweli S, Meyer E, Okoh A (2018). Antibiotic use in agriculture and its consequential resistance in environmental sources: Potential public health implications. Molecules.

[44] Kanfer R, Ackerman PL (1989). Motivation and cognitive abilities: An integrative/aptitude^treatment interaction approach to skill acquisition. J. Appl. Psychol..

[45] WHO, Europe (2019). The Fight Against Antimicrobial Resistance: Benefits from Behavioural Insights.

[46] Schreijer A, van de Sande-Bruinsma N, den Daas C, Lo Fo Wong D (2014). Tailoring AMR strategies (TAP): When knowledge is not enough. Eur. J. Public Health..

[47] DLS (2020). One Health in Action: DLS Initiatives on AMR/AMU.

[48] Moyen N (2018). A large-scale study of a poultry trading network in Bangladesh: Implications for control and surveillance of avian influenza viruses. BMC Vet. Res..

[49] Gupta SD, Hoque MA, Fournié G, Henning J (2020). Patterns of Avian Influenza A (H5) and A (H9) virus infection in backyard, commercial broiler and layer chicken farms in Bangladesh. Transbound. Emerg. Dis..

[50] Beaubien JM (2000). Principles and practice of structural equation modeling. Pers. Psychol..

[51] Ajzen I (1985). Action Control: From Cognition to Behaviour.

[52] Hu L-T, Bentler PM (1999). Cutoff criteria for fit indexes in covariance structure analysis: Conventional criteria versus new alternatives. Struct. Equ. Model..

[53] Browne MW, Cudeck R (1992). Alternative ways of assessing model fit. Sociol. Methods Res..

[54] Pituch KA, Stevens JP (2015). Applied Multivariate Statistics for the Social Sciences: Analyses with SAS and IBM’s SPSS.

