# Supplementary material for: Social and cognitive factors influencing commercial chicken farmers’ antimicrobial usage in Bangladesh
Source: Sci Rep. 2023 Jan 11;13:572. doi: 10.1038/s41598-022-26859-8 (PMC9834256; doi:10.1038/s41598-022-26859-8)
Supplement: Supplementary file 1 — Supplementary Information. [file 41598_2022_26859_MOESM1_ESM.docx]

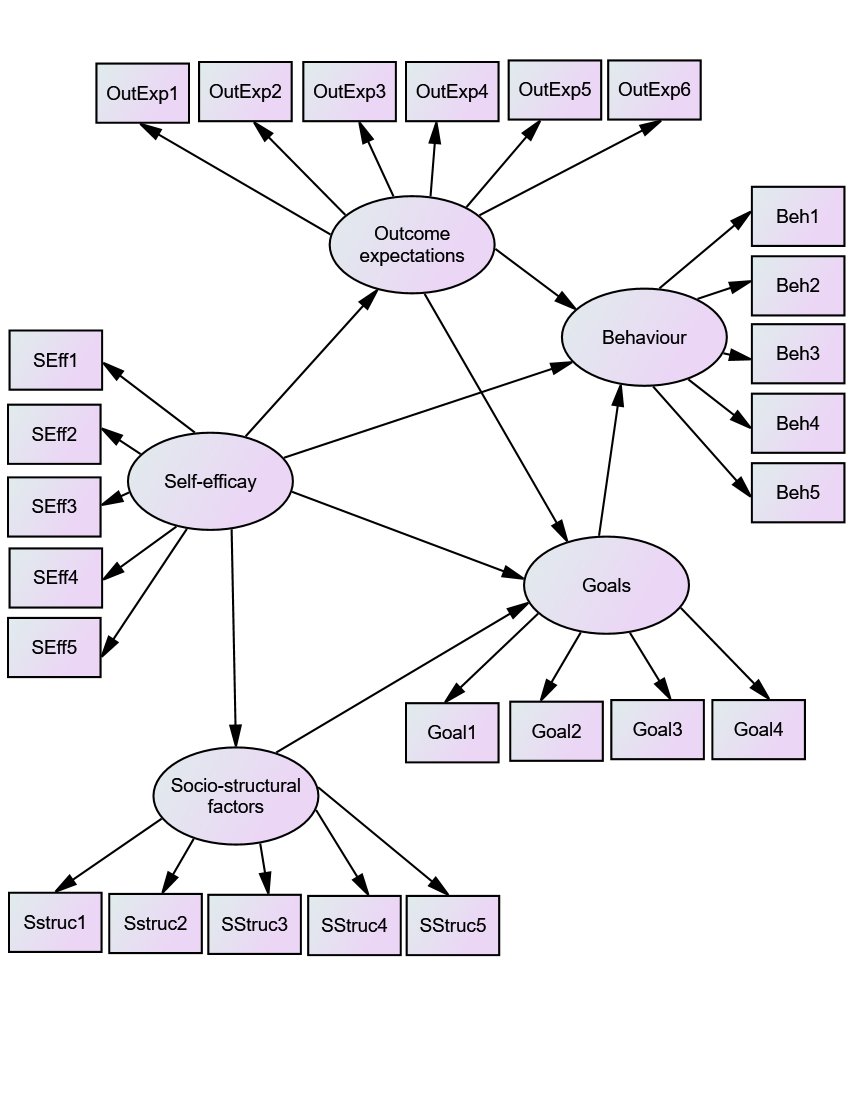


**Figure S1.** Conceptualized SEM path model describing farmers’ *behaviour* towards antimicrobial application on commercial chicken farms in Chattogram, Bangladesh. Observed variables (statements) are presented in rectangles and the latent constructs are presented in ovals. Both ‘d’ and ‘e’ represents errors of measurements for items while ‘z’ represents the residuals of the latent constructs.

*SEff1-SEff5* *represent the statements under the latent construct Self-efficacy (Table S1)*

*OutExp1-OutExp6 represent the statements under the latent construct Outcome expectations (Table S1)*

*Sstruc1-Sstruc5 represent the statements under the latent construct Socio-structural factors (Table S1)*

*Beh1-Beh5 represent the statements under the latent construct Behaviour (Table S1)*

*Goal1-Goal4 represent the statements under the latent construct Goals (Table S1)*

| Statement | Strongly disagree | Disagree | Do not know | Agree | Strongly agree |
| --- | --- | --- | --- | --- | --- |
|  | **% (N)** | **% (N)** | **% (N)** | **% (N)** | **% (N)** |
| *Behaviour* |  |  |  |  |  |
| I am increasing the dosage of antimicrobial when I am experiencing more chicken getting sick or dying (Beh1) | 5.1 (7) | 43.8 (60) | 0.0 (0) | 48.9 (67) | 2.2 (3) |
| I like to use multiple antimicrobial products to treat my chickens (Beh2) | 3.6 (5) | 48.2 (66) | 0.7 (1) | 46.0 (63) | 1.5 (2) |
| I always have a range of antimicrobial available on my farm, even if don’t used them all (Beh3) | 5.8 (8) | 61.3 (84) | 0.0 (0) | 32.8 (45) | 0.0 (0) |
| I am increasing the dosage of antimicrobial when egg production decreases/ broilers do not grow fast enough (Beh4) | 8.0 (11) | 78.1 (107) | 1.5 (2) | 11.7 (16) | 0.7 (1) |
| It is difficult to find time to maintain good chicken health, so providing antimicrobial help me to save my valuable time (Beh5) | 2.9 (4) | 66.4 (91) | 5.8 (8) | 22.6 (31) | 2.2 (3) |
| *Outcome expectations* | | | | | |
| Antimicrobials can be used in chickens any dosage, route and frequency (OutExp1) | 7.3 (10) | 70.1 (96) | 1.5 (2) | 21.2 (29) | 0.0 (0) |
| Antimicrobial residues in chicken meat will not harm humans (OutExp2) | 3.6 (5) | 28.5 (39) | 19.7 (27) | 45.3 (62) | 2.9 (4) |
| Antimicrobial provided to chickens will be destroyed during cooking and frying (OutExp3) | 4.4 (6) | 2.2 (3) | 10.9 (15) | 72.3 (99) | 10.2 (14) |
| Antimicrobial can be given to chickens up to the day before selling (OutExp4) | 8.0 (11) | 60.6 (83) | 3.6 (5) | 27.0 (37) | 0.7 (1) |
| If an antimicrobial has healed my chickens once, it will always be effective to heal the same disease in the future (OutExp5) | 5.1 (7) | 70.1 (96) | 7.3 (10) | 17.5 (24) | 0.0 (0) |
| If antimicrobial is used in chicken, it will stay in chickens and would not be excreted (OutExp6) | 3.6 (5) | 60.6 (83) | 24.1 (33) | 11.7 (16) | 0.0 (0) |
| *Self-efficacy* | | | | | |
| I am skilled enough to select and administer antimicrobial without anyone’s advice (SEff1) | 1.5 (2) | 24.8 (34) | 0.0 (0) | 66.4 (91) | 7.3 (10) |
| I would invest time to participate in training on the proper use of antimicrobials (SEff2) | 2.2 (3) | 11.7 (16) | 2.9 (4) | 64.2 (88) | 19.0 (26) |
| I believe that stronger laws and enforcement of the law are needed to reduce antimicrobial usage (SEff3) | 0.0 (0) | 0.0 (0) | 5.1 (7) | 47.4 (65) | 47.4 (65) |
| I would invest time and money to further improve farm hygiene and biosecurity to reduce the usage of antimicrobial on my farm (SEff4) | 0.0 (0) | 10.9 (15) | 4.4 (6) | 64.2 (88) | 20.4 (28) |
| I am not sure what to do when someone gives me advice about the usage of antimicrobial in chickens (SEff5) | 6.6 (9) | 68.6 (94) | 4.4 (6) | 19.7 (27) | 0.7 (1) |
| *Goals* | | | | | |
| Antimicrobial leads to a healthy growth of chickens (Goal1) | 3.6 (5) | 55.5 (76) | 2.2 (3) | 32.8 (45) | 5.8 (8) |
| Antimicrobial helps chickens to recover from disease (Goal2) | 0.0 (0) | 0.0 (0) | 0.7 (1) | 87.6 (120) | 11.7 (16) |
| Antimicrobial helps increase the egg production/ improve the quality of the chicken meat (Goal3) | 5.1 (7) | 73.0 (100) | 11.7 (16) | 8.0 (11) | 2.2 (3) |
| Antimicrobial use in chickens will improve human health (Goal4) | 66.4 (40) | 1.5 (2) | 29.2 (40) | 2.2 (3) | 0.7 (1) |
| *Socio-structural factors* | | | | | |
| Antimicrobials are expensive to buy (SStruc1) | 0.0 (0) | 2.2 (3) | 0.0 (0) | 57.7 (79) | 40.1 (55) |
| There is a lack of clear national guidelines on how to use antimicrobials in chickens (SStruc2) | 0.7 (1) | 13.9 (19) | 32.8 (45) | 44.5 (61) | 8.0 (11) |
| The labels of antimicrobial products do not provide clear information on how to use the antimicrobial (SStruc3) | 6.6 (9) | 47.4 (65) | 8.0 (11) | 36.5 (50) | 1.5 (2) |
| I am bound to take advice from feed dealers because I owe them money (they provide day old chicks, antimicrobials, and feed) (SStruc4) | 7.3 (10) | 67.9 (93) | 4.4 (6) | 19.7 (27) | 0.7 (1) |
| Antimicrobials and vaccines are same products (SStruc5) | 0.7 (1) | 19.7 (27) | 4.4 (6) | 68.6 (94) | 6.6 (9) |

**Table S1.** Percentage (N) of responses to statements (‘observed variables’) provided by commercial layer and broiler chicken farmers in Chattogram, Bangladesh. Using the Social Cognitive Theory framework, responses explaining each of ‘latent variables’ (*outcome expectations*, *self-efficacy, behaviour*, *goals*, and *socio-structural factors*) considered in the analysis. *Behaviour* of using antimicrobials was used as an outcome variable.
